# OPN/CD44v6 overexpression in laryngeal dysplasia and correlation with clinical outcome

**DOI:** 10.1038/sj.bjc.6604070

**Published:** 2007-11-06

**Authors:** S Staibano, F Merolla, D Testa, R Iovine, M Mascolo, V Guarino, M D Castellone, M Di Benedetto, V Galli, S Motta, R M Melillo, G De Rosa, M Santoro, A Celetti

**Affiliations:** 1 Dipartimento di Scienze Biomorfologiche e Funzionali, Università di Napoli ‘Federico II’, Naples, Italy; 2 Dipartimento di Biologia e Patologia Cellulare e Molecolare, Università di Napoli ‘Federico II’, Istituto di Endocrinologia ed Oncologia Sperimentale del CNR, Naples, Italy; 3 Clinica Otorinolaringoiatrica, Seconda Università di Napoli, Naples, Italy; 4 Dipartimento Assistenziale di Otorinolaringoiatria e Scienze Affini, Università di Napoli ‘Federico II’, Naples, Italy

**Keywords:** OPN, SCC, dysplasia, marker

## Abstract

Laryngeal dysplasia is a common clinical concern. Despite major advancements, a significant number of patients with this condition progress to invasive squamous cell carcinoma. Osteopontin (OPN) is a secreted glycoprotein, whose expression is markedly elevated in several types of cancers. We explored OPN as a candidate biomarker for laryngeal dysplasia. To this aim, we examined OPN expression in 82 cases of dysplasia and in hyperplastic and normal tissue samples. OPN expression was elevated in all severe dysplasia samples, but not hyperplastic samples, with respect to matched normal mucosa. OPN expression levels correlated positively with degree of dysplasia (*P*=0.0094) and negatively with disease-free survival (*P*<0.0001). OPN expression was paralleled by cell surface reactivity for CD44v6, an OPN functional receptor. CD44v6 expression correlated negatively with disease-free survival, as well (*P*=0.0007). Taken as a whole, our finding identify OPN and CD44v6 as predictive markers of recurrence or aggressiveness in laryngeal intraepithelial neoplasia, and overall, point out an important signalling complex in the evolution of laryngeal dysplasia.

Laryngeal squamous cell carcinoma (LSCC) is the most common type of head and neck squamous cell carcinoma (HNSCC). LSCC accounts for 1–2% of all malignancies diagnosed worldwide (Vokes *et al*, 1993; Licitra *et al*, 2003; Mao *et al*, 2004). Notwithstanding primary prevention, screening, surgical treatment, and radiotherapy, the long-term survival rate of LSCC patients has remained substantially unchanged in the last two decades (Hoffman *et al*, 1998). Survival of the patients depends on the stage of the disease; therefore, early detection and timely therapy are essential (Vokes *et al*, 1993; Hoffman *et al*, 1998; Licitra *et al*, 2003; Mao *et al*, 2004).

Laryngeal squamous cell carcinoma usually develops in a multistep process: normal mucosa – dysplasia (laryngeal intraepithelial neoplasia, LIN) – LSCC *in situ* – invasive LSCC (Rosai *et al*, 1992; Tabor *et al*, 2002; Zuckerberg, 2002; Johnson, 2003). Dysplasia is characterised by increased cell growth, cellular atypia (nuclear and nucleolar abnormalities, altered nuclear/cytoplasmatic ratio, and altered cytoplasmatic differentiation), and architectural alteration of the epithelium. Conventionally, the dysplastic changes are graded as mild (LIN I: dysplasia limited to the basal third of the epithelium, few mitoses), moderate (LIN II: dysplasia involving the lower two-thirds of the epithelium, marked nuclear changes, prominent nucleoli, mitoses in the parabasal, and intermediate layers), and severe (LIN III: dysplasia involving more than two-thirds of the epithelial thickness, nuclear pleomorphism and hyperchromasia, prominent nucleoli, cell crowding, and atypical mitoses). Often, severe dysplasia and *in situ* carcinoma are grouped in the same category (Rosai *et al*, 1992; Tabor *et al*, 2002; Zuckerberg, 2002; Johnson, 2003). Early forms of dysplasia may be reversible if the initial stimuli (like smoke and volatile irritating substances) are removed, while severe dysplasia, if left untreated, is regarded as a precancerous lesion (Rosai *et al*, 1992; Tabor *et al*, 2002; Zuckerberg, 2002; Johnson, 2003). For patients with mild or moderate dysplasia, the reported rate of progression to invasive cancer is up to 11.5 and 45%, respectively. In severe dysplasia, higher rates of progression are commonly reported (Rosai *et al*, 1992; Tabor *et al*, 2002; Zuckerberg, 2002; Johnson, 2003). The molecular events that induce the evolution of dysplasia to carcinoma are still unknown (Cowan *et al*, 1992; Zuckerberg, 2002; Perez-Ordonez *et al*, 2006).

Osteopontin (OPN), also known as SPP1 (secreted phosphoprotein 1), is a highly acidic calcium-binding glycosylated phosphoprotein (Weber, 2001; Rittling and Chambers, 2004; Rangaswami *et al*, 2006). OPN can function both as cell adhesion molecule and as cytokine. It binds to the cell surface receptors *α*v- or *β*1-containing integrins and CD44v6 (Weber, 2001; Rittling and Chambers, 2004; Rangaswami *et al*, 2006), thereby supporting proliferation, chemotaxis, attachment, and migration of many cell types. CD44 is a cell surface glycoprotein that is involved in regulating cell–cell and cell–matrix interactions, migration, and tumour growth and progression (Ponta *et al*, 2003). CD44 is expressed as a standard receptor (CD44s) and in multiple splice isoforms (CD44v), whose expression is altered during tumour growth and progression. Expression of the ‘v6’ variant exon of CD44 is necessary for OPN binding (Ponta *et al*, 2003). OPN is overexpressed in many human tumours, for example, colon, breast, liver, prostate, gastric, ovarian, lung, thyroid, and kidney carcinomas (Agrawal *et al*, 2002; Kang *et al*, 2003; Ye *et al*, 2003; Schorge *et al*, 2004; Donati *et al*, 2005; Guarino *et al*, 2005; Matusan *et al*, 2006).

We recently reported that OPN and CD44v6 are overexpressed in full-blown LSCC (Celetti *et al*, 2005). Here, we have investigated the role of the OPN/CD44v6 axis in laryngeal dysplasia.

## MATERIALS AND METHODS

### Study population

Patients (82 cases: 77 men and 5 women) underwent surgery at the Otolaryngology Department of the University Federico II of Naples between January 1993 and December 2001. The patients' age ranged from 23 to 83 years, with a mean of 62.46 years. Paraffin blocks were retrieved from the files of the Department of Biomorphological and Functional Sciences, Pathology Section, University Federico II of Naples. Each patient agreed to and signed a consent for the treatment of clinical data and tissues for diagnostic and research purposes, according to the guidelines of the Institutional Ethic Committee. For all the patients, clinicopathologic and follow-up data were recorded (Table 1). Before surgery, patients underwent otolaryngological, fibreoptic, and radiological evaluation. The mean follow-up time was 10.1 years (range: 8–13 years). Follow-up consisted in clinical and radiological evaluation at 3-month intervals for the first year and 6-months intervals thereafter. At completion of follow-up, patients were subdivided into alive with absence of relapse (no); alive with evidence of recurrent disease (LIN); and alive with progression of disease to LSCC (SCC).

After surgical resection, tissues were fixed in 10% neutral buffered formalin and embedded in paraffin blocks. Sections (4-*μ*M thick) were stained with haematoxylin–eosin for histological examination. The pathologic analysis was performed in a blinded fashion to the clinical informations. The cytological evaluation was according to standard criteria (Evans *et al*, 1986; Gale *et al*, 2000; Tabor *et al*, 2003).

### Immunohistochemistry

Four-micromolar thick serial sections, mounted on poly-L-lysine-coated glass slides, were dewaxed, rehydrated through multiple graded ethanol solutions, treated with 3% hydrogen peroxide for 5 min to inactivate endogenous peroxidases, and washed in distilled water. After antigen retrieval (microwave oven 5 min × 3 times, in 1% citrate buffer), nonspecific binding was blocked by incubation (2 h at room temperature) with 1.5% blocking serum. Slides were first incubated with anti-OPN (final concentration: 5 *μ*g ml^−1^) (10A16; Assay Designs, Ann Arbor, MI, USA) or anti-CD44v6 (dilution of 1 : 100) (NCL-CD44v6, clone VFF-7; Novocastra Laboratories Ltd, Newcastle upon Tyne, UK) monoclonal antibodies and then with biotinylated anti-IgG and the premixed avidin–biotin complex (overnight at 4°C) (Vectastain ABC kits; Vector Laboratories, Burlingame, CA, USA). The immune reaction was revealed with 0.06 mmol l^−1^ diaminobenzidine (DAB-DAKO, Carpinteria, CA, USA) and 2 mmol l^−1^ hydrogen peroxide. Finally, slides were counterstained with haematoxylin and coverslipped with a synthetic mounting media. Control slides in the presence of preimmune serum were included for each staining as an additional negative control. Anti-OPN antibody was preincubated with a fivefold molar excess of OPN peptide to ascertain specificity of the reaction.

The results of the immunohistochemical staining were evaluated separately and in a blinded fashion by two pathologists. Five representative microscopic areas at × 400 magnification were randomly selected for examination. Expression of OPN was semiquantitatively assessed as percentage of positive cells with respect to the total number of epithelial cells. The samples were assigned to one of the four following categories: 0 (absence of positive cells); + (<10% of positive cells); ++ (10–50% of positive cells); and +++ (>50% of positive cells). Staining of CD44v6 was classified as ‘lower’ (lower third) (L), ‘lower and middle’ (up to two-thirds of the thickness of the epithelium) (M), and ‘full thickness’ (F).

### Statistical analysis

The Pearson's *χ*^2^ test was used to assess the statistical significance of the frequency distribution of all categories of OPN or CD44v6 expression by degree of dysplasia or by relapse. Differences were significant with *P*-value <0.05.

Nonparametric Spearman's correlation coefficient method was used to assess the statistical significance of the correlation between OPN expression *vs* CD44v6 positivity. A test was run for all the patients’ cohort combined or grouped by degree of dysplasia or by type of relapse. Correlations were significant when *P*-value was <0.05. Disease-free survival curves of the patients were calculated using the Kaplan–Meier method, and analysis was performed by the log-rank test. Differences were significant when *P*<0.05. In this analysis, a group of 31 patients has been censored for lack of data. Statistical analysis was performed using the JMP software program (version 5.1.1; SAS Institute Inc., Austin, TX, USA).

## RESULTS

### Immunohistochemical detection of OPN and CD44v6 in laryngeal dysplasia

**Figure 1:**
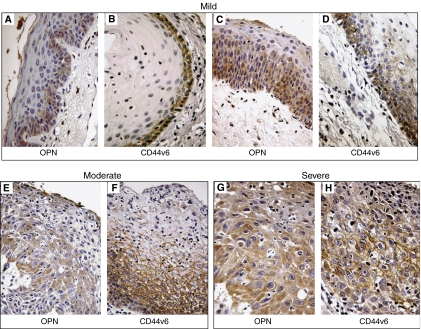
(**A** and **B**) A representative case of mild degree dysplasia showing weak immunostaining for OPN (**A**) and CD44v6 positivity restricted to the lower third of the epithelium (**B**) ( × 150). (**C** and **D**) Another mild dysplasia sample showing stronger immunostaining for both the markers ( × 150). (**E** and **F**) A representative case of moderate dysplasia showing a strong immunostaining for OPN (**E**) and a CD44v6 positivity involving the lower two-thirds of the epithelium (**F**) ( × 250). (**G** and **H**) A case of high-grade dysplasia showing strong immunostaining for OPN (**G**) and CD44v6 positivity up to the upper third of the epithelium (**H**) ( × 400).

Eighty-two laryngeal samples with different degree of dysplasia (Table 1) and the matched normal mucosa were tested for OPN expression by immunohistochemistry with an anti-OPN-specific monoclonal antibody. Representative stainings are shown in Figure 1, and the entire data set is reported in Table 2. OPN was virtually undetectable (<2.0% of the cells) in normal tissue (*n*=10). Dysplastic areas showed different degrees of OPN positivity. In most (76%) of mild dysplasia cases, only few cells (+) were positive (Figure 1A), while 50% of moderate and severe dysplasia samples had intense (+++) OPN staining (Figures 1E and G). Only 20% cases of mild dysplasia were highly positive for OPN (+++); interestingly, in these samples, OPN expression coexisted with a diffuse CD44v6 staining (see below) (Figures 1C and D).

The samples were also analysed for the expression of CD44v6, the receptor that is involved in OPN binding (Table 2). Only basal cells (L category) were CD44v6 positive in 76% of mild dysplasia samples (Figure 1B). Instead, in moderate dysplasia, CD44v6 positivity was found in the basal two-thirds (M category) (Figure 1F) or even full thickness (F category) of the epithelium. Finally, 50% of severe dysplasia samples showed a full-thickness (F category) CD44v6 staining (Figure 1H). The association between OPN and CD44v6 immunoreactivity resulted significant when analysed by the Spearman's rank correlation test (Table 3A). The frequency distribution of OPN positivity or CD44v6 immunoreactivity by degree of dysplasia resulted highly significant at Pearson's *χ*^2^ test (Table 4A).

**Figure 2:**
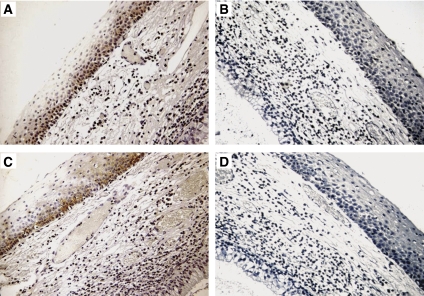
(**A** and **C**) A representative case of mild degree dysplasia showing weak immunostaining for OPN (**A**) and CD44v6 positivity restricted to the lower third of the epithelium (**C**) ( × 150). (**B** and **D**) The same samples as in (**A**) negative for OPN immunostaining after incubation with isotype control antiserum (**B**) ( × 150), and the same sample as in (**C**) negative for CD44v6 immunostaining after incubation with isotype control antiserum (**D**) ( × 150).

Foci of squamous metaplasia of laryngeal cylindric-cell-lined areas were almost constantly present in our samples. Metaplastic areas (*n*=20) were almost constantly negative for OPN and CD44v6 staining (Figures 2A and B). Only in few (5%) cases, we observed an intense (+++) OPN staining paralleled by full-thickness CD44v6 positivity in the squamous metaplastic cells (Figures 2C and D). Although the ultimate statistical relevance of this finding is still to be verified on larger series of cases, it is interesting to note that these OPN- and CD44v6-positive metaplasia areas were found in patients with a history of development of SCC at the follow-up.

### OPN and CD44v6 expression levels in laryngeal dysplasia negatively correlate with disease-free survival

**Figure 3:**
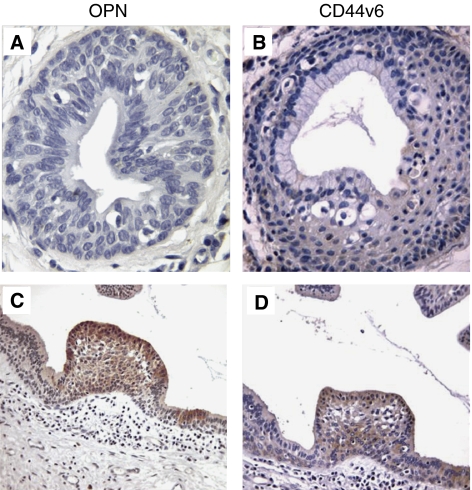
(**A** and **B**) An area of incomplete squamous metaplasia, negative for OPN (**A**), and CD44v6 (**B**) immunostaining ( × 250). (**C** and **D**) Another case of incomplete squamous metaplasia (patient with a history of laryngeal SCC), showing an intense OPN (**C**) and CD44v6 (**D**) immunostaining ( × 106).

**Figure 4:**
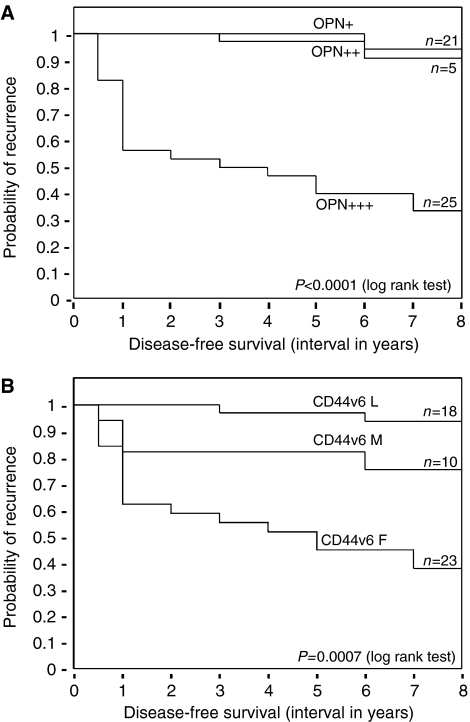
Osteopontin and CD44v6 staining in laryngeal dysplasia negatively correlates with disease-free survival. Kaplan–Meier survival plots for patients grouped on the basis of the OPN (**A**) or CD44v6 (**B**) expression level. (**A**) Patients were stratified into three categories (+ (*n*=21), ++ (*n*=5), and +++ (*n*=25)) based on OPN immunostaining intensity. (**B**) Patients were stratified into three categories (L (*n*=18), M (*n*=10), and F (*n*=23) based on the thickness of CD44v6 staining). The *P*-value was determined by a two-sided log-rank test.

The disease-free survival rate in patients affected by laryngeal dysplasia negatively correlated with intense OPN staining and full-thickness CD44v6 positivity. As shown by the Kaplan–Meier survival curves reported in Figure 3A, the 8-years disease-free survival was 94 and 91% for OPN (+)- and OPN (++)-positive cases, respectively, and 33% for OPN (+++)-positive cases (two-sided log-rank test, *P*<0.0001; Figure 4A). Relative to CD44v6 expression, the probability of recurrence was 94 and 75% for cases that showed basal (L), or basal and middle (M) staining, respectively, and 38% for patient that had full-thickness (F) positivity (two-sided log-rank test, *P*=0.007; Figure 4B).

At the Pearson's test, the frequency distribution of OPN and CD44v6 expression levels were significantly correlated with relapse (Table 4B).

Moreover, the correlation between OPN and CD44v6 expression in patients with absence of relapse, with recurrence of dysplasia, or with progression to LSCC resulted very significant at the Spearman's rank correlation test (Table 3B). Finally, the contingency analysis showed that the frequency distribution of OPN by CD44v6 were highly significant in combined or grouped types of relapse (Table 4C).

## DISCUSSION

An in-depth understanding of the factors involved in the initial steps of LSCC development will facilitate the prevention and diagnosis of this condition. Currently, histological grading and the topographical extension of laryngeal dysplasia are used to predict the risk for cancer and to determine the treatment strategy (Rosai *et al*, 1992; Zuckerberg, 2002; Johnson, 2003). Although significant efforts have been made to identify molecular markers of the clinical outcome of premalignant laryngeal lesion, still neither single nor combination of markers is accepted in clinical practice (Tabor *et al*, 2002).

We have previously reported that both OPN and CD44v6 are highly expressed in invasive LSCC (Celetti *et al*, 2005). Here, we have explored whether the same ligand/receptor pair is involved in the premalignant phases as well. To this aim, we have investigated OPN and CD44v6 in a set of laryngeal dysplasia samples and correlated their expression level to histological grading and clinical outcome. Both OPN and CD44v6 were consistently overexpressed in dysplastic but not hyperplastic or metaplastic mucosa. Dysplasia, in particular high-grade dysplasia, is regarded as a preneoplastic condition (Rosai *et al*, 1992). Importantly, OPN/CD44v6 overexpression was significantly correlated with the degree of dysplasia, type of recurrence, and reduced disease-free survival. Taken together, these findings suggest that OPN signalling through CD44v6 may play a role in the establishment of dyplastic changes in the laryngeal epithelium. Interestingly, it has been recently reported that genetic deletion of OPN in transgenic mice did not change the rate of hyperplasia formation but caused a reduction of benign papilloma formation after the two-stage skin chemical carcinogenesis protocol; thus, also in this experimental model system, OPN is involved in the early phases of tumorigenesis (Hsieh *et al*, 2006).

Osteopontin is able to engage several cell surface receptors, including integrins and CD44 variants. In particular, OPN binds CD44 proteins that contain v6-encoded sequences, and OPN/CD44v6 binding has been implicated in carcinogenesis (Ponta *et al*, 2003). Here, we show that OPN expression levels were paralleled by intense expression of CD44v6; at contingency analysis, the frequency distribution of OPN expression by CD44v6 positivity resulted highly significant at Pearson's test; moreover, the association between OPN and CD44v6 immunoreactivity was highly significant at the Spearman's correlation coefficient test, suggesting that CD44v6 is at least one of the functional OPN receptors in laryngeal dysplasia.

To investigate whether the OPN/CD44v6 overexpression was causally related with dysplasia, cytological changes induced by OPN stimulation of primary human keratinocytes, obstructed by CD44 blockade, have been observed (Celetti A *et al*, in preparation). Addressing CD44 as a functional receptor for OPN would be important to explore the molecular mechanism underlying dysplastic changes induced by the OPN/CD44 axis. It is known that CD44 triggering stimulates diverse signalling pathways, including activation of ERK (Bourguignon *et al*, 2005), RAC (Teramoto *et al*, 2005), and RHO (Bourguignon *et al*, 2003), as well as secretion of soluble factors, like cytokines and metalloproteinases (Zhang *et al*, 2002; Bourguignon *et al*, 2003; Murphy *et al*, 2005). These pathways are potentially involved in dysplastic changes induced by OPN/CD44v6.

A model for the initiation and progression of colorectal cancer has become a paradigm for other human solid tumours (Fearon and Vogelstein, 1990). Like colorectal cancer, HNSCC is thought to progress through a series of well-defined clinical and histopathological stages. While not all of the specific mutations required for progression have been delineated, a working molecular model has been proposed (Silverman, 2003). The loss of chromosomal regions 3p and 9p21 are among the first identified genetic changes (Mao *et al*, 1996; Sanz-Ortega *et al*, 2003). In particular, loss-of-heterozygosity (LOH) at 9p21 in conjunction with promoter hypermethylation results is the inactivation of the *CDKN2A* gene, coding for the cyclin-dependent kinase inhibitor 2A (p16INK4). This alteration occurs prior to the development of histologic atypia and is associated with the transition from normal to hyperplastic/metaplastic mucosa (Papadimitrakopoulou *et al*, 2001; Sanz-Ortega *et al*, 2003). Subsequent LOH at 17p with mutation of the *TP53* tumour suppressor gene is associated with progression to dysplasia (Boyle *et al*, 1993). The overexpression of the EGF receptor is also an early event in carcinogenesis (Rubin Grandis *et al*, 1998). Amplification and overexpression of the *CCND1* gene, encoding cyclin D1 is a common late event in HNSCC formation (Michalides *et al*, 1995; Izzo *et al*, 1998; Chatrath *et al*, 2006). Our findings suggest that the upregulation of the OPN/CD44v6 axis is an additional early event during the progression of laryngeal dysplasia. Thus, early immunocytochemical detection of OPN and CD44v6 can be exploited to set a screening test for laryngeal dysplasia. Moreover, perturbation of OPN/CD44v6 signalling may represent a promising novel strategy to prevent progression of laryngeal preneoplastic lesions.

## Figures and Tables

**Table 1:** Clinicopathological features of studied laryngeal intraepithelial neoplasia (LIN) patients

**Characteristics**	**Total (%)**
*No. of subjects*	
Male	77 (94)
Female	5 (6)
	
*Disease site*	
Glottis–hypoglottis	43 (53)
Supraglottis	39 (47)
	
*Degree of dysplasia*	
Mild	21 (47)
Moderate	8 (19)
Severe	53 (64)
	
*Relapse*	
No	35 (43)
LIN	10 (12)
SCC	37 (45)

SCC=squamous cell carcinoma.

**Table 2:** OPN and CD44v6 positivity in laryngeal intraepithelial neoplasia at different degree

**LIN**	**OPN positivity^a^**	**CD44v6 positivity^a^**
Mild (21)	16/21 (+)	16/21 (L)
	1/21 (++)	2/21 (M)
	4/21 (+++)	3/21 (F)
Moderate (8)	3/8 (+)	2/8 (L)
	1/8 (++)	3/8 (M)
	4/8 (+++)	3/8 (F)
Severe (53)	16/53 (+)	15/53 (L)
	11/53 (++)	12/53 (M)
	26/53 (+++)	26/53 (F)

OPN=osteopontin.

a Osteopontin and CD44v6 expression were assessed by immunohistochemistry and scored respectively as follows: +=<10% positive cells; ++=10–50% positive cells; and +++=50–100% positive cells; L=lower; M=lower and middle; and F=full-thickness involvement of the epithelial layers.

**Table 3A:** Correlation of osteopontin and CD44v6 expression in all laryngeal intraepithelial neoplasia (LIN) patients combined or grouped by degree of dysplasia

**LIN**	***r*_s_**	***P*_s_**
Combined (82)	0.8231	<0.0001
Mild (21)	0.9941	<0.0001
Moderate (8)	0.2622	=0.5304
Severe (53)	0.7957	<0.0001

Note: Correlation between osteopontin and CD44v6 expression in LIN patients analysed by Spearman's rank correlation test.

Correlation coefficient (*r*_s_) and *P*_s_ are shown (*P*_s_<0.05 was considered significant).

**Table 3B:** Correlation of osteopontin and CD44v6 expression in all laryngeal intraepithelial neoplasia (LIN) patients combined or grouped by relapse

**Relapse**	***r*_s_**	***P*_s_**
Combined (82)	0.8231	<0.0001
no (35)	0.8133	<0.0001
LIN (10)	0.5976	=0.0734
SCC (37)	−0.7892	<0.0001

SCC=squamous cell carcinoma.

Note: Absence of relapse (no), recurrence of dysplasia (LIN), progression to carcinoma (SCC).

Correlation between osteopontin and CD44v6 expression in LIN patients analysed by Spearman's rank correlation test. Correlation coefficient (*r*_s_) and *P*_s_ are shown (*P*_s_<0.05 was considered significant).

**Table 4:** Pearson's test

	***χ* ^2^**	***P***
*A. Contingency analysis of osteopontin (OPN) and CD44v6 positivity by degree of dysplasia*		
OPN	13.425	0.0094
CD44v6	16.198	0.0028
		
*B. Contingency analysis of OPN and CD44v6 positivity by relapse (no, LIN, SCC)*		
OPN	21.780	<0.0002
CD44v6	9.567	0.0484
		
*C. Contingency analysis of OPN positivity by CD44v6 expression in all laryngeal intraepithelial neoplasia combined or grouped by relapse*		
Combined (82)	73.026	<0.0001
No (35)	32.694	<0.0001
LIN (10)	10.000	0.0067
SCC (37)	34.857	<0.0001

LIN=laryngeal intraepithelial neoplasia; SCC=squamous cell carcinoma.

Note: Absence of relapse (no), recurrence of dysplasia (LIN), and progression to carcinoma (SCC).
